# Plasma protein signatures altered before and after tuberculosis diagnosis in a population-based cohort

**DOI:** 10.1186/s12014-026-09602-7

**Published:** 2026-06-06

**Authors:** Natalia Koziar, Anthony D. Whetton, Nophar Geifman

**Affiliations:** 1 School of Biosciences, Faculty of Health and Medical Sciences, University of Surrey, Guildford, GU2 7XH UK; 2 Veterinary Health Innovation Engine, School of Veterinary Medicine, University of Surrey, Guildford, GU2 7AL UK; 3 School of Health Sciences, Faculty of Health and Medical Sciences, University of Surrey, Guildford, GU2 7YH UK

**Keywords:** Tuberculosis, Plasma Proteomics, Differential Expression Analysis, UK Biobank

## Abstract

Tuberculosis (TB) continues to be a major public health concern. Thus, identifying individuals at risk of progression to active disease is important for community health but remains difficult. To address this issue, we analysed plasma proteomic data from the UK Biobank to investigate host protein signatures associated with TB. The size of the prospective cohort (~ 500,000) combined with deep proteomic analysis (2,920 plasma proteins) allowed us to perform analysis on 42 individuals diagnosed with TB either before or after sampling, alongside matched controls with no history of TB. Using this approach, we identified significantly differentially expressed proteins (e.g. CDCP1, GAST) in individuals affected by TB, suggesting proteomics alternations that precede clinical onset and persist years after diagnosis. Functional annotation of significant proteins indicated involvement of immune and signalling pathways associated with chronic inflammatory responses and host defence mechanisms. These findings help advance the understanding of TB pathology and offer the potential for use of specific plasma proteins in the development of point of care risk assessment and diagnostic tools.

**Supplementary Information:**

The online version contains supplementary material available at 10.1186/s12014-026-09602-7.

## Introduction

Tuberculosis (TB), caused by the bacterium *Mycobacterium tuberculosis*, remains a major global health concern. In 2023, TB was estimated to have caused 10.8 million new infections and 1.25 million deaths, making it the leading cause of mortality from a single infectious agent [1]. Despite the fact that approximately 25% of the global population are believed to be infected with the responsible bacterium, only approximately 5–10% of those infected will go on to develop TB in their lifetime, making a positive test for *Mycobacterium tuberculosis* infection alone a poor indicator of future TB risk [2, 3]. Therefore, further research into potential biomarkers is desperately required, but obtaining sufficient numbers of TB samples and datasets remains challenging.

It is known that inflammation is a hallmark of TB due to the bacteria’s survival strategies within host cells triggering a prolonged inflammatory response [4]. This activity consequently alters the host’s proteome, impacting various cellular pathways and processes to favour the pathogen’s viability and replication [5, 6]. As a result, over the past decades, host biomarkers such as antibodies and cytokines have begun to be explored as potential diagnostic markers for both latent and active TB, with certain proteins delivering promising results in terms of accuracy and sensitivity but limited through a lack of validation studies [7, 8]. Furthermore, the complex host-pathogen interaction makes it difficult to pinpoint reliable biomarkers, many of which are not specific to *M.tuberculosis* infection but are instead general markers of inflammation [9].

In this study, we used data from the UK Biobank to compare the expression of 2,920 plasma proteins between individuals diagnosed with active TB prior to blood sample collection, those diagnosed after sampling and matched healthy controls (never diagnosed with TB). Proteins discovered to be significantly differentially expressed between participants afflicted with TB and controls were then functionally annotated to provide insight into biological processes and pathways involved in TB pathology.

## Methods

### Study participants

Data was obtained from the UK Biobank database, a large-scale prospective study consisting of 503,317 participants aged 40–69 years at baseline. Proteomic analysis was carried out on a subset of blood samples collected at recruitment, which included 46,673 randomly selected participants, and 6,385 selected by the UK Biobank Pharma Proteomics Project (UKB-PPP) consortium. At the time of analysis, 40 participants had withdrawn consent, resulting in 53,018 individuals with available proteomics.

The proteomic cohort was then filtered for the International Classification of Disease version 10 (ICD10) codes corresponding to *M. tuberculosis* infection (A15-A19), identifying 50 participants, of which 26 were diagnosed with TB after blood sample collection at recruitment, and 24 diagnosed with TB prior to enrolment and sample collection. The exclusion of ICD10 codes for *M. tuberculosis infection*, alongside several other respiratory and autoimmune conditions (acute and chronic lower respiratory conditions, human immunodeficiency virus, rheumatic fever, arthritis, systemic lupus erythematosus), were then used to filter for a control cohort, identifying a pool of 33,422 participants. All respective ICD10 codes are available in Supplementary Table 1.

### Proteomics

Circulating plasma proteins were identified using the Olink Explore platform across four panels (Cardiometabolic I, Inflammation I, Neurology I, Oncology I), resulting in the measurements of 2,923 unique proteins. Details of sample preparation, processing, and quality control have been previously described [10]. Three proteins (GLIPR1, NPM1, PCOLCE) that had > 30% missingness across all samples were removed, alongside participants with > 20% missingness across the remaining protein measurements, resulting in a control cohort of 27,949 participants and a TB cohort of 42 participants. Remaining missing values were imputed using half of the lowest value present in the dataset for each respective protein.

### Statistical analysis

Every case of TB was matched to 5 control cases using propensity score matching based on age, sex, and Townsend deprivation index through the ‘MatchIt’ package (version 3–7.0) in R (version 4.5.5) using nearest neighbour matching. Differential expression protein analysis was performed with the ‘limma’ package (version 3.54.2) between matched controls, those diagnosed with TB prior to blood sampling, and those diagnosed after. As Olink NPX values are already on a log2 scale, no additional log transformation was applied, and the resulting log2 fold changes therefore represent differences in mean NPX values between groups. The criteria for significantly expressed proteins were determined with a Benjamini–Hochberg adjusted p-value threshold of < 0.05 and a log2 fold change threshold of > 0.5, with complete results available in Supplementary Tables 3–7.

## Results

The plasma profiles of 23 individuals diagnosed with TB prior to sample collection, 19 individuals diagnosed after sample collection, and 210 controls were measured using an antibody-based affinity assay, targeting a total of unique 2,920 proteins. Time of diagnosis relative to sampling at initial assessment ranged from 12 years prior to 12 years after, with most participants diagnosed within 7 years of the date of initial assessment (Supplementary Fig. 1). The characteristics of the 252 study participants are further described in Table 1, with full definition, exclusion criteria and comorbidities available in Supplementary Tables 1 and 2.

**Table 1:** Study participant demographics and clinical characteristics

	**TB (All)**	**TB (Before Sample)**	**TB (After Sample)**	**Control**	**P Value**
**Number of Participants**	42	23	19	210	
**Age (Years)**					0.42*1*^*a*^
**Range**	41–67	41–67	42–67	40–69	
**Mean**	54.3	52.8	56.1	54.0	
**Sex**					0.733^b^
**Female**	16	8	8	91	
**Male**	26	15	11	119	
**Deprivation Index**					0.892^a^
**Range**	−4.52–7.44	−4.52–7.43	−3.83–7.44	−5.09–10.2	
**Mean**	0.860	0.666	1.10	1.03	
**Time to Diagnosis relative to Sampling (Years)**					
**Range**	−12.3–12.8	−12.3 – −0.096	0.986–12.8		
**Mean**	−0.658	−6.15	5.99		

^a^ calculated from one-way ANOVA test

^b^ calculated from x^2^ test

**Fig. 1:**
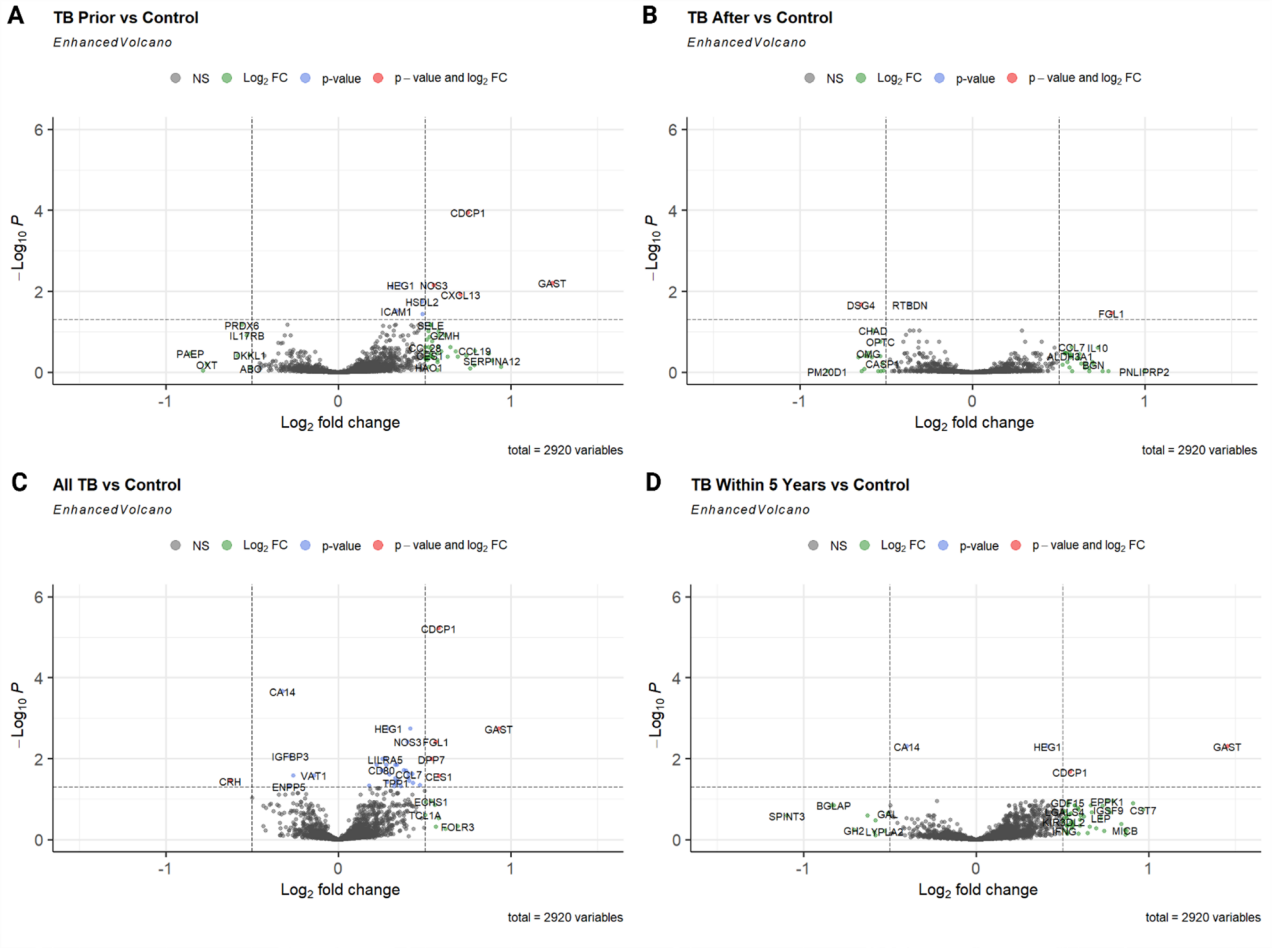
Differential expression analysis. **(A)** Differentially expressed proteins in 23 participants diagnosed with TB prior to blood sampling versus 115 matched controls, **(B)** differentially expressed proteins in 19 participants diagnosed with TB after blood sampling versus 95 matched controls, **(C)** all 42 participants diagnosed with TB versus 210 controls, **(D)** 19 participants diagnosed with TB within 5 years of sampling versus 95 matched controls. Models were adjusted for sex, age and Townsend deprivation index. Statistically significant proteins were determined using a log2 fold change threshold of > 0.05 and Benjamini–Hochberg adjusted p-value of < 0.05

Differential expression analysis was performed between those diagnosed with TB prior or after sampling, those diagnosed with TB at any time (‘All TB’), and matched controls (Fig. 1A-C and Supplementary Tables 3–6). Additionally, we carried out sensitivity analysis by including only participants diagnosed with TB 5 years within their initial assessment (Fig. 1D and Supplementary Table 7). Statistically significant proteins were determined using a log2 fold change (FC) threshold of > 0.5 and a Benjamini–Hochberg adjusted p-value of < 0.05. Proteins significantly associated with tuberculosis showed varied expression patterns relative to time of diagnosis from sampling (Supplementary Fig. 2).

A total of 4 proteins: CUB Domain Containing Protein 1 (CDCP1), Gastrin (GAST), Nitric Oxide Synthase 3 (NOS3) and C-X-C Motif Chemokine Ligand 13 (CXCL13) were found to be significantly upregulated in participants diagnosed with TB prior to blood sampling when compared to controls. When comparing participants diagnosed with TB after sampling to controls, 2 proteins were significantly differentially expressed: Fibrinogen-like protein 1 (FGL1) was upregulated in participants afflicted with TB, while Desmoglein 4 (DSG4) was downregulated. 5 proteins: CDCP1, GAST, FGL1, Dipeptidyl Peptidase 7 (DPP7) and Carboxylesterase 1 (CES1) were significantly upregulated in all participants diagnosed with TB when compared to controls, and 1 protein: Corticotropin-Releasing Hormone (CRH) was significantly downregulated. Limiting the analysis to participants diagnosed with TB within 5 years of sampling only, showed that 2 proteins (GAST and CDCP1) remained significantly upregulated in participants afflicted with TB. No proteins were significantly differentially expressed when comparing participants diagnosed with TB prior to sampling to those diagnosed after.

**Table 2:** Functional annotation of significantly differentially expressed proteins using the Database for Annotation, Visualization, and Integrated Discovery (DAVID)

Protein	Contrast(s)	GO Biological Process	KEGG Pathway
CES1	All TB	Cholesterol homeostasis, lipid metabolic process, reverse cholesterol transport, cholesterol metabolic process, cellular response to cholesterol	Drug metabolism - other enzymes
CRH	All TB	Inflammatory response, signal transduction, positive regulation of cAMP/PKA signal transduction, hormone-mediated apoptotic signaling pathway, response to xenobiotic stimulus	cAMP signaling pathway
DPP7	All TB	Proteolysis, lysosomal protein catabolic process	No returned pathways
GAST	TB Prior, All TB	Signal transduction, G protein-coupled receptor signaling pathway	Hormone signaling, gastric acid secretion
CDCP1	TB Prior, All TB	No returned terms	No returned pathways
NOS3	TB Prior	Nitric oxide mediated signal transduction, nitric oxide biosynthetic process, lipopolysaccharide-mediated signaling pathway, vasodilation, cell redox homeostasis	Arginine and proline metabolism, HIF-1 signaling pathway, PI3K-Akt signaling pathway, VEGF signaling pathway
CXCL13	TB Prior	Chronic inflammatory response, defence response to bacterium, chemokine-mediated signaling pathway, regulation of humoral immune response, neutrophil chemotaxis	Cytokine-cytokine receptor interaction, chemokine signaling pathway,
FGL1	TB After, All TB	Adaptive immune response, negative regulation of T cell activation, blood coagulation, hepatocyte proliferation	No returned pathways
DSG4	TB After	Cell adhesion, keratinocyte differentiation, BMP signalling pathway	Cornified envelope formation

Where a large number of GO or pathway terms were returned for a given protein, representative terms relevant to TB pathophysiology were selected to minimise redundancy

Functional annotation of significantly differentially expressed proteins using DAVID (Table 2) revealed a range of Gene Ontology (GO) terms and KEGG pathways across comparisons between different TB clinical states and matched controls. Proteins differentially expressed in participants diagnosed with TB prior to sampling are shown to be implicated in functions such as immune activation, signalling, and vascular modulation. Meanwhile, proteins differentially expressed in participants diagnosed after sampling are involved in processes such as immune regulation and tissue remodelling. Proteins differentially expressed when comparing all TB participants against controls, show involvement in functions such as intracellular signalling, lipid metabolism and homeostasis. For some proteins, such as CDPC1 and DPP7, functional characterisation was limited due to a current lack of GO or KEGG annotations.

## Discussion

In this study, we leveraged proteomic data from the UK Biobank to compare the plasma protein profiles of participants diagnosed with TB either prior to or after blood sampling, against matched controls with no history of TB or other common respiratory or autoimmune conditions. Our results revealed both distinct and overlapping proteomic signatures associated with TB and the diagnosis timeframe, suggesting that host plasma proteins can capture biological signals reflective of TB pathology.

Despite participants unlikely to have active TB when a blood sample was taken, a differential proteomic signature could still be observed when compared to matched controls. Participants diagnosed prior to blood sampling showed statistically significant up-regulation of NOS3 and CXCL13, possibly indicating a lasting host response years after diagnosis and/or despite treatment. Macrophages infected with *M. tuberculosis* have been shown to induce NOS3 expression, with recent work describing nitric oxide (NO) to play a central role in driving the physiological changes that lead to differentially culturable phenotypes of *M. tuberculosis* associated with persistence and treatment tolerance [11, 12]. CXCL13 is required for the formation and maintenance of organized lymphoid structures and granulomas during *M. tuberculosis* infection, possibly reflecting the persistent immune response observed in patients after antitubercular treatment [13, 14]. Participants diagnosed after sampling showed downregulation of DSG4 and upregulation of FGL1, potentially demonstrating early host responses or susceptibility factors present before clinically apparent disease. Recent studies have demonstrated DSG4 is significantly downregulated in the serum of patients with cavitary pulmonary TB; a severe form of the disease characterised by the destruction of lung tissue [15]. FGL1 is a major inhibitory ligand of Lymphocyte Activation Gene 3 (LAG-3), a checkpoint receptor that is upregulated on T cells during tuberculosis and contributes to suppression of effector T-cell function [16, 17]. Upregulation of FGL1 prior to clinical diagnosis, therefore, may reflect the early activation of immune regulatory pathways during subclinical disease.

Two proteins; GAST and CDCP1, remained significantly differentially expressed when restricting the analysis to diagnoses within 5 years of sampling only. Both proteins were upregulated in individuals afflicted with TB, compared to controls. While GAST has not been previously associated with TB in current literature, pro-inflammatory cytokines known to be elevated in active tuberculosis, such as Tumour Necrosis Factor alpha (TNF-α), have been shown to directly stimulate gastrin expression and secretion [18, 19]. The function of CDCP1 is not well described, however, it has been reported in other studies exploring plasma protein signatures associated with tuberculosis [20, 21]. CDCP1 can act as a ligand for CD6 to activate T cells [22], therefore, an elevated expression of these proteins may be reflecting a systemic inflammatory state associated with *M. tuberculosis* infection.

Our study has several limitations. First, results were generated from a relatively small sample size, albeit that this number of proteins has never been quantified in blood from TB patients before. Furthermore, the UK Biobank cohort while fairly reflective of the UK population, is less likely to include subgroups at higher risk of TB in the UK, such as those living in more socioeconomically deprived areas [23]. To address potential selection bias of those with a TB diagnosis, comparator groups were selected by matching controls based on demographics including the Townsend deprivation index. Second, microbiological confirmation of TB was not available for every participant, therefore making it impossible to rule out the possibility that an individual was misdiagnosed. Third, although we employed methods to control for cofounding variables such as age, sex, and deprivation index, the presence of unidentified confounding factors cannot be ruled out. Lastly, the cross-sectional nature of the proteomic data limits our ability to draw conclusions about temporal changes or causality; post-diagnosis samples likely reflect treatment and immune recovery, potentially blunting or altering proteomic signals. Additionally, we cannot exclude the possibility that residual or prolonged immune activation from factors such as incomplete treatment or post-infectious immune dysregulation, may have contributed to the proteomic signals observed in those diagnosed before sampling.

Our findings demonstrate that plasma proteomic profiling can identify biologically meaningful signatures associated with tuberculosis both before and after clinical diagnosis, highlighting the potential of proteomics for uncovering host biomarkers relevant to TB pathogenesis. Further validation in larger, independent cohorts with microbiologically confirmed diagnoses will be required to validate these findings.

## Supplementary Information

Supplementary Material 1.

## Data Availability

This research was conducted using the data from the UK Biobank with the application number 83988. UK Biobank data is available on application to the UK Biobank (www.ukbiobank.ac.uk/).

## Abbreviations

TB: Tuberculosis
UKB-PPP: UK Biobank Pharma Proteomics Project
ICD-10: International Classification of Disease version 10
FDR: False discovery rate
CDCP1: CUB domain containing protein 1
GAST: Gastrin
NOS3: Nitric Oxide Synthase 3
CXCL13: C-X-C motif chemokine ligand 13
FGL1: Fibrinogen-like protein 1
DSG4: Desmoglein 4
DPP7: Dipeptidyl Peptidase 7
CES1: Carboxylesterase 1
CRH: Corticotropin-Releasing Hormone
NO: Nitric Oxide
LAG-3: Lymphocyte Activation Gene 3
TNF-α: Tumour Necrosis Factor alpha
